# Understanding the Neural Basis of Prospective Memory Using Functional Near-Infrared Spectroscopy

**DOI:** 10.3389/fnhum.2022.905491

**Published:** 2022-06-16

**Authors:** Yu Wen Koo, David L. Neumann, Tamara Ownsworth, Michael K. Yeung, David H. K. Shum

**Affiliations:** ^1^School of Applied Psychology, Griffith University, Mt Gravatt, QLD, Australia; ^2^The Hopkins Centre, Menzies Health Institute of Queensland, Griffith University, Gold Coast, QLD, Australia; ^3^Department of Rehabilitation Sciences, The Hong Kong Polytechnic University, Kowloon, Hong Kong SAR, China

**Keywords:** prospective memory (PM), fNIRS (functional near infrared spectroscopy), prefrontal cortex, BA10, young adults

## Abstract

Prospective memory (PM) is the ability to perform a planned action at an intended future time. This study examined the neural correlates of PM using functional near-infrared spectroscopy (fNIRS). This study employed a within-participants design. A laboratory PM task was adapted for use with fNIRS to investigate regions of interest and levels of brain activation during task performance in 32 participants (63% female, *M_age_* = 21.31 years, *SD_age_* = 4.62 years). Participants first completed a working memory (WM) task (N-back ongoing task) followed by a WM plus PM task while neural activity was measured using fNIRS. Behavioral results revealed an interference effect for reaction time on the WM task, whereby participants were significantly slower to respond in the WM plus PM task compared to the WM task. Ongoing task accuracies did not differ between the two conditions. fNIRS results revealed a higher level of neural activity in the fronto-polar prefrontal cortex and dorsolateral prefrontal cortex in the WM plus PM task compared to the WM Condition. These findings highlight that fNIRS is a suitable tool for studying and understanding the neural basis of PM.

## Introduction

Prospective memory (PM) is the ability to perform a planned action at an intended time in the future. It is a complex process involving at least four stages: encoding, storage, delayed retrieval, and enactment of intended actions (Ellis, 1996; Kliegel et al., 2002). PM ability is crucial to independent living across the lifespan. Examples of PM in daily life include remembering to attend appointments or delivering a message to a friend. Importantly, PM is distinct from other neurocognitive abilities like retrospective memory (e.g., recalling or recognizing past events or previously learned information) and executive function (e.g., updating, inhibition, and switching) at the conceptual, neuropsychological, and neurobiological levels (see Gupta et al., 2010).

To date, most research on PM has been behavioral. In particular, most studies were designed to identify important variables that affect PM performance such as cue focality, task order (Kliegel et al., 2008; Ihle et al., 2013), and to compare PM performances between age groups (Henry et al., 2004; Aberle et al., 2010; Kliegel et al., 2016; Koo et al., 2021) and clinical populations (Ramanan and Kumar, 2013; Zhou et al., 2019). Meanwhile, understanding the neural basis of PM processes has received comparatively less attention and most studies used fMRI. Thus, the aim of the study was to evaluate the suitability of fNIRS for investigating PM and provide corroborating evidence for the neural basis of PM using this technique.

Evidences from early neuroimaging studies have typically found changes in the blood-oxygen level-dependent (BOLD) signal or increases in regional cerebral blood flow within the rostral prefrontal cortex (i.e., BA10) when comparing event-based PM performance to ongoing task only (Okuda et al., 1998; Burgess et al., 2001, 2003). This pattern of activation appears relatively independent of the type of stimuli used, the level of difficulty in detecting the PM cue, the nature of the ongoing task, or whether PM targets are actually encountered (Burgess et al., 2001, 2003; Simons et al., 2006). Other regions commonly activated during event-based PM (as compared to ongoing task-only) include co-activations inthe supramarginal gyrus (i.e., BA7) and posterior cortex such as BA40 (Burgess et al., 2011). Thus far, current evidence suggests that PM processes involve highly localized prefrontal areas. A meta-analysis by Cona et al. (2015) found that the dorsalfrontoparietal network and ventral front oparietal networks are involved in PM maintenance and PM retrieval, respectively.

In PM research, the use of functional near-infrared spectroscopy (fNIRS) is still in its infancy. To date, only a few studies have used fNIRS to investigate PM. The first study investigated ecological PM tasks using a fiberless, wearable fNIRS in a single participant (Pinti et al., 2015). A healthy 24-year-old was asked to remember to respond to a PM cue (e.g., a familiar face or a parking meter) while performing an ongoing task. There were four conditions: uncontaminated ongoing condition (walk around the experimental areas and count the number of signs affixed to buildings containing the word *Queen*), non-social PM condition (count number of dates and opening hours on buildings, but also touch parking meters if they were nearby), social PM condition (count number of doorbells and fist-bump the researcher within the area), and an additional contaminated ongoing condition (count number of unobstructed stairways within the area). Results showed increases in oxy-Hb and decreases in deoxy-Hb in response to PM hits (i.e., greater activation when responding to PM cues compared to the ongoing task) in the PFC. They also found different patterns of activation when acting on a non-social vs. social PM cue. Despite being a preliminary single-case study, which only examined activities in the overall PFC, this study illustrated the ecological validity and feasibility of using fNIRS to monitor PFC activity during a real-world PM task which mimicked activities in everyday life.

Subsequently, Dong et al. (2017) assessed PM using fNIRS in an immersive virtual reality (VR) environment in 11 healthy young participants (*M*_age_ = 25.4 years, *SD* = 25.4). The ongoing task and PM tasks were performed in a shopping street with 12 shops, two special action points, and an exit. Participants used a joystick and body movements to navigate the virtual streets. For the ongoing task, participants were instructed to press a button when passing each store and read aloud a number under the store signboard. The PM task required participants to purchase items from a shopping list and perform specific actions. Their results showed that activation in BA10 during the PM component was significantly greater compared to the ongoing component, which is consistent with previous fMRI neuroimaging studies (e.g., Burgess et al., 2011).

Subsequently, Dong et al. (2019) compared neural activity using fNIRS between a laboratory-based PM task and the same VR PM task from their previous study (Dong et al., 2017). The study included 10 healthy young male participants (*M*_age_ = 22.5 years, *SD* = 22.5). Their laboratory-based PM task involved judging which two numbers presented were larger using the corresponding keys (left and right). For the PM task, participants were to press the “up” key when the numbers were of equal value. They replicated their previous study, showing that BA10 was significantly more active during the PM component compared to the ongoing component in their VR task. However, they also found that hemodynamic changes of BA10 in the VR PM task were greater than those in the laboratory-based PM task. In addition, when comparing the VR PM task and the laboratory-based task, they found that the VR task induced greater hemodynamic changes across both the ongoing and PM component. The authors postulated that these observed differences could be due to the nature of the VR PM task, as it resembles a more realistic environment than a conventional laboratory-based task.

Together, the studies by Pinti et al. (2015) and Dong et al. (2017, 2019) support the feasibility of using fNIRS to investigate brain activities related to PM performance across both task types. However, a limitation of these studies are that they included small sample sizes (*N* < 11), and it is unclear what laboratory-based paradigms are compatible with the fNIRS methodology. Moreover, the first case study (Pinti et al., 2015) did not map out sub-brain areas as a region of interest. Meanwhile, Dong et al. (2017, 2019) only collected data from male participants, which is not representative of the general population. Therefore, further investigation into the study of PM using fNIRS using a representative sample with a larger sample size is warranted. In this study, we adopted an *n*-back task with PM cues embedded to form a classic dual-task paradigm (Einstein and McDaniel, 1990) as the PM task. This was then used to compare cerebral activity and behavioral performance to the *n*-back task without PM cues.

In sum, fNIRS is an emerging neuroimaging technique that could help further elucidate the neural basis of PM. The aims of this study were to: (1) develop an experimental PM task (i.e., dual-task paradigm) that is suitable to investigate neural activation during PM processes using fNIRS; and (2) provide corroborating evidence for the neural basis of PM. We compared the behavioral data and fNIRS parameters between an ongoing task [Working Memory (WM) only] and when a PM task was added (WM plus PM). Based on the results of previous neuroimaging studies, it was hypothesized that rostrolateral PFC (BA10), dorsolateral PFC, and frontopolar cortex (Gilbert et al., 2009; Basso et al., 2010) would show greater activations during the WM plus PM Condition compared to the WM Condition.

## Materials and Methods

### Participants

There were 32 participants (63% female, *M_age_* = 21.31 years, *SD_age_* = 4.62 years). They were all first-year university psychology undergraduates at Griffith University who participated for course credit. All participants had a normal or corrected-to-normal vision, and no history of neurological or psychiatric diseases. The experiment was approved by the Griffith University Ethics Committee (GU Ref: 2019/1002). All participants were informed about the procedure and operating mode of the fNIRS prior to providing written consent.

### Design

This experiment employed a within-participant design (Task Condition: WM Condition vs. WM plus PM) for the WM and PM parameters (RT, accuracy). fNIRS data were continuously recorded across both conditions.

### Materials

#### Ongoing and PM Tasks

For the WM Condition, participants were presented with a 2-back letter working-memory task as an ongoing task (adapted from West et al., 2006). Target stimuli for the 2-back task were capital letters (B, C, F, G, H, J, L, M, R, V, X, Z, and X) in 18-point sized font and a viewing distance of 60 cm surrounded by rectangular borders in one of 10 different colors (olive, yellow, cyan, red, purple, magenta, gray, green, blue, and maroon). Participants had to decide whether the letter presented for a trial occurred two trials ago, by pressing the “1” key for Yes and the “2” key for No. Letters were presented for 500 ms, followed by a blank screen which allowed a response up to 2,000 ms before a fixation cross appeared for 500 ms. The inter-stimulus interval was 3,000 ms. At the beginning of the WM Condition, participants were asked to complete a practice block consisting of 20 trials (seven WM target trials, 13 non-target). This was followed by two blocks of 36 trials per block (i.e., 12 WM target trials and 24 non-target trials). The dependent variables were the total number of correct responses for the ongoing task trials and the average RT of the correct responses for the ongoing task.

**Figure 1:**
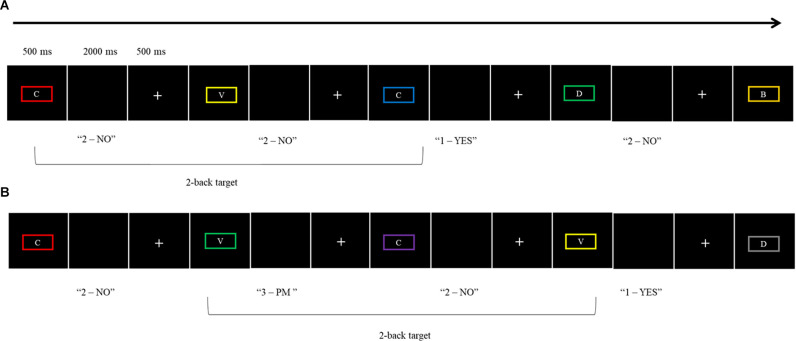
Schematic illustration of the two experimental conditions: **(A)** WM Condition, **(B)** WM plus PM Condition.

The WM plus PM Condition was introduced after the WM condition to avoid contamination. The PM task was embedded into the ongoing task (i.e., the 2-back task) to form a classic dual-task paradigm (Einstein and McDaniel, 1990). For the PM task, participants were instructed to press the “3” key rather than the ongoing task response keys of 1 or 2 when the border surrounding the letter was green. There were five PM cues in each of the two blocks (a total of 10 PM cues). Like the WM Condition, participants first completed a practice block consisting of 20 trials for the WM plus PM Condition (two PM cues, eight WM target trials, and 10 non-target trials). This was followed by two blocks of 36 trials. The dependent variables for this condition were PM accuracy (proportion correct), PM average RT, WM ongoing task accuracy (proportion correct), and WM ongoing average RT. See Figure 1 for the schematic illustration of both the WM Condition and WM plus PM Condition.

#### Cognitive Measures

##### Wechsler Abbreviated Scale of Intelligence

The Wechsler Abbreviated Scale of Intelligence—second edition (WASI-II) is a short form IQ test designed to estimate intelligence in adults and older adolescents (ages 6–89 years). Two subscales (i.e., vocabulary and matrix reasoning) were used to calculate estimated full-scale IQ (FSIQ-2). In an adult sample, the average internal consistency reliability coefficient for FSIQ-2 is 0.93 (McCrimmon and Smith, 2012).

##### Letter-Number Sequencing Subtest

The Letter Number Sequencing Test (LNS) is a subtest of the Wechsler Memory Scale (WMS-III; Wechsler, 1997). Participants were presented with a series of numbers and letters, and asked to recall the numbers in numerical order followed by the letters in alphabetical order. A series of alternating numbers and letters at the rate of about 1 per second was orally presented. Participants were instructed to first report the numbers in ascending numerical order, then letters in alphabetical order. The test begins with a series of two items (one number and one letter) and continues to a maximum of eight items (four numbers and four letters). Participants were given three trials at each series length and continued until all three trials of a series length failed. The maximum possible score for Letter-Number Sequencing is 21. Test-retest reliability was found to be between 0.71 and 0.77 (Wechsler, 2008), with high internal consistency of 0.85 (Gold et al., 1997).

##### Prospective and Retrospective Memory Questionnaire

The Prospective and Retrospective Memory Questionnaire (PRMQ; Smith et al., 2000) is a 16-item questionnaire developed to measure the frequency of prospective (PM) and retrospective (RM) memory failures in everyday life. Half the questions measure PM (e.g., Do you decide to do something in a few minutes time and then forget to do it?), while the other half measures RM failures (e.g., Do you forget what you watched on television the previous day?). Participants are required to rate how often each type of memory failure happens in their everyday life on a 5-point scale ranging from very often (5) to never (1). The overall reliability of PRMQ is considered high with Cronbach’s alpha ranging from 0.72 (Zeintl et al., 2006) to 0.92 (Crawford et al., 2006). A score for PM and RM in addition to the total score can be calculated by totaling questions for each subscale with higher scores indicating more frequent everyday PM and RM failures.

##### BAPM

The Brief Assessment of Prospective Memory (BAPM; Man et al., 2011) is a 16-item self-report questionnaire originally designed to assess the frequency of PM failures for individuals with traumatic brain injury. Participants are required to rate their PM forgetting in the last month on a 5-point scale from 1 (never), 2 (rarely), 3 (occasionally), 4 (often), 5 (very often), or NA (not applicable). The ratings were made for each of eight instrumental activities of daily living (IADL) items such as managing finances, shopping, and meal preparation, and eight basic activities of daily living (BADL) items such as eating, dressing, and personal grooming. Part A is a 16-item self-reported questionnaire that assesses PM failures within the last month. Three average scores are calculated from this scale—the total overall PM, BADL subscale, and IADL subscale scores. In a healthy sample, reliability for the BAPM total score ranged from 0.84 to 0.83 indicating acceptable internal consistency (Man et al., 2011).

### Procedure

**Figure 2:**
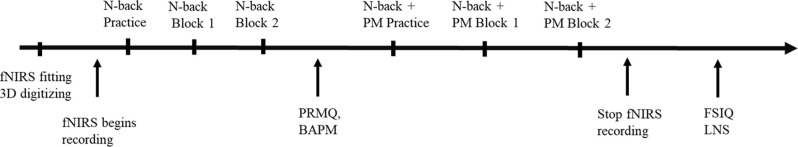
Schematic illustration of the procedural flow for experiment 1.

All participants were informed about the aim of the experiment upon arrival and provided written consent before the fNIRS cap was fitted. Participants first performed the practice trial and two blocks of WM Condition (ongoing task). After that, they were administered the practice trial and two blocks of the WM plus PM task (PM task) to avoid “contamination” of the ongoing task performance (Simons et al., 2006; Gilbert et al., 2009). They were given a short rest period after each task block (30 s fixation cross-screen post-task and 30 s fixation cross-screen pre-task). All stimuli were presented using E-Prime 3.0 software (Psychology Software Tools, Pittsburgh, PA). Each task block lasted about 7.5 min. Participants completed the self-reported measures between the WM Condition and WM plus PM Condition. Participants completed the cognitive measures (i.e., FSIQ-2 and LNS) after the fNIRS recording stopped. The experiment took approximately 1 h to complete. See Figure 2 for the procedural flow of the experiment.

#### fNIRS Data Collection

**Figure 3:**
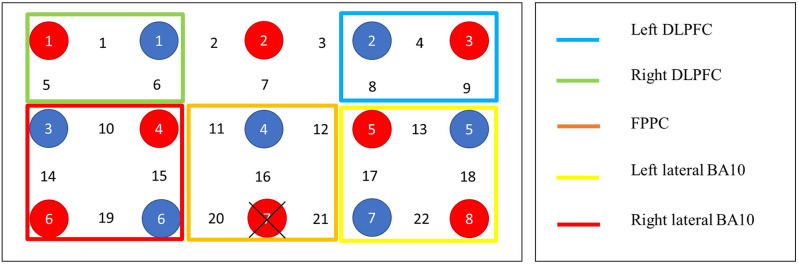
Probe layout and channel groupings. Note. Distance between each source and detector represent 3 cm. Red circles denote detectors, blue circles denote emitters. Fpz marked at the cross.

The fNIRS recording session was completed in a quiet, dimly-lit room. Each participant sat approximately 60 cm from a 15” monitor displaying the experimental task. A Shimadzu LABNIRS continuous-wave 24 channel fNIRS system was used to collect data during the experimental tasks. This system operates at three wavelengths (790, 805, and 830 nm) measured with a time resolution of 135 ms. In accordance with the International 10–20 system, a tight fitting fNIRS cap was used to position eight emitter and eight detector probes 3 cm apart across the frontal lobes (see Figure 3). The anatomical locations of optodes were determined for each participant in relation to standard head landmarks including inion; nasion; top center (Cz); and left and right tragi using a Patriot 3D Digitizer (Polhemus, Colchester, VT). Prior to data collection, the strength and quality of channel signals on the fNIRS machine was tested. Any channels displaying attenuation values less than 60 db or greater than 140 db were removed, cleaned, and reconnected to ensure there is no excess noise or interference. Data were continuously recorded and marked *via* trigger cables as a block commenced and ended. Changes in the concentration of total Hb (sum of oxy- and deoxy-Hb) and hemoglobin difference (HbD; oxy- minus deoxy-Hb) was calculated as a measure of changes in cerebral blood flow (Tsuji et al., 1998).

#### Statistical Analyses

All data were analyzed using SPSS 27 (IBM Corporation, NY, USA) and the alpha level was set at 0.05. All continuous variables followed a normal distribution with kurtosis and skewness values between −1.5 and +1.5. The data were screened for accuracy, missing values, outliers, and normality. Given the directional nature of the hypothesis, one-tailed paired *t*-tests were conducted to compare the effect of the Task Conditions (WM Condition vs. WM plus PM) on the parameters of the WM and PM DVs. An alpha level of 0.05 was used for all tests.

#### fNIRS Data Analyses

fNIRS data were preprocessed using Homer3 (Huppert et al., 2009). For the (oxy-Hb) data, bad channels were removed, then a Butterworth low-pass filter with a half power frequency of 0.10 Hz was applied to remove abrupt movement artifact before converting the raw intensity data to optical density changes based on the modified Beer-Lambertlaw. Next, correlation-based signal improvement (CBSI) was applied to correct for motion artifacts (Cui et al., 2010). This is based on the negative correlation between the dynamics of (oxy-Hb) and (deoxy-Hb), this transforms the time courses of (oxy-Hb) and (deoxy-Hb) into mirror images of each other. Hence, only changes in the CBSI-corrected (oxy-Hb) were analyzed. A temporal window was cut from −30 to 290 s relative to the onset of blocks (*t* = 0 s) for averaging. The time course of (oxy-Hb) from 10 to 109 s was averaged to obtain the mean (oxy-Hb) induced by the conditions.

A block averaging method was employed. Signal segments across task vs. rest periods were averaged, inferring functional brain activity based on the difference between task and rest mean values (for review, see Pinti et al., 2020). A block protocol was employed to average across sets of trials, with each trial consisting of a pre-task resting baseline period and an event period that began at stimulus onset. The resting baseline was calculated as the mean oxy-Hb across from −10 s to the 0 s prior to stimulus presentation and the event period was calculated as the average across the entire active Block 1 and Block 2 (0–109 s per block).

#### Optode Localization

A region of interest (ROI) approach was applied using a 3D digitizer and Atlas Viewer (Aasted et al., 2015) to approximately localize the NIRS channels on a standardized template. Due to our task including both WM and PM components, we included PFC ROIs since both constructs have been shown to localize in prefrontal regions such as the bilateral rostrolateral prefrontal areas, frontopolar cortex, and dorsolateral PFC (Owen et al., 2005; Basso et al., 2010; Rottschy et al., 2012). However, given that the focus of our study was on PM, the ROI for PM was biased to BA10, which is congruent with prior studies investigating event-based PM (Schroeter et al., 2002; Okuda et al., 2007). Accordingly, five ROIs were identified: left dorsolateral prefrontal cortex (DLPFC), right DLPFC, fronto-polar prefrontal cortex (FPPC), left-lateral, and right lateral PFC (BA10) following Tsuchiya et al. (2017), who used the same fNIRS machine. Figure 3 shows the probe layout and channel groupings. Owing to the lower spatial resolution of NIRS and lack of structural guidance imagery, it is challenging to precisely map activity to brain regions. Thus, the specified ROIs were treated as approximations and interpreted with caution (Ozawa et al., 2019).

## Results

All 32 participants were included in the final analyses as they performed at a satisfactory level (i.e., at least 50%) for the WM ongoing task. See Table 1 for means and *SD*s on sociodemographic and cognitive measures and there were no outliers for the cognitive variables.

**Table 1:** Mean scores and standard deviations of sociodemographic and cognitive variables (*N* = 32).

	*M*	*SD*
Age (years)	21.31	4.62
Years of education	12.97	1.23
FSIQ-2	100.19	7.32
LNS	9.12	2.17
BAPM Part A IADL	2.18	0.58
BAPM Part A BADL	1.57	0.40
BAPM Part A Total	1.87	0.44
PRMQ RM	18.63	4.38
PRMQ PM	21.56	5.42
PRMQ Total	40.19	9.02

*Note: FSIQ-2, Full Scale Intellectual Quotient from Wechsler Abbreviated Scale of Intelligence-II; LNS, Letter Number Sequencing; BAPM, Brief Assessment of Prospective Memory; IADL, Instrumental Activities of Daily Living; BADL, Basic Activities of Daily Living; PRMQ, Prospective and Retrospective Memory Questionnaire*.

### Behavioral Results

To evaluate whether WM task accuracy and RT differed according to task condition, we conducted one-tailed paired *t*-tests to compare the WM Condition and WM plus PM Condition. See Table 2 for means and *SDs* for task accuracies and RTs.

**Table 2:** Mean RTs (ms) and accuracy (proportion correct) for task conditions in ongoing WM and PM task parameters.

	WM Condition		WM plus PM		PM only		WM Condition vs. WM plus PM Condition		
DV	*M*	*SD*	*M*	*SD*	*M*	*SD*	*t*_(31)_	*p*	*d*
Accuracy (Proportion Correct)	0.85	0.08	0.86	0.10	0.68	0.25	−0.54	0.267	−0.10
RT (ms)	682	182	820	267	782	266	−4.25	<0.001	−0.75

Paired *t*-tests revealed that there was no significant differences between the WM task accuracies for the WM Condition and WM plus PM Condition, *t*_(31)_ = −0.54, *p* = 0.267, *d* = −0.10. For WM task RTs, there was a significant difference in RT between WM Condition and WM plus PM Condition, *t*_(31)_ = −4.25, *p* < 0.001, *d* = −0.75, which was a medium to large effect size. As shown in Table 2; a significantly longer mean RT was observed in the WM plus PM Condition than in the WM Condition. For PM accuracy there was no ceiling no floor effect (*M* = 0.68, *SD* = 0.25), with average PM RT of 782 ms (*SD* = 266).

### fNIRS Results

**Figure 4:**
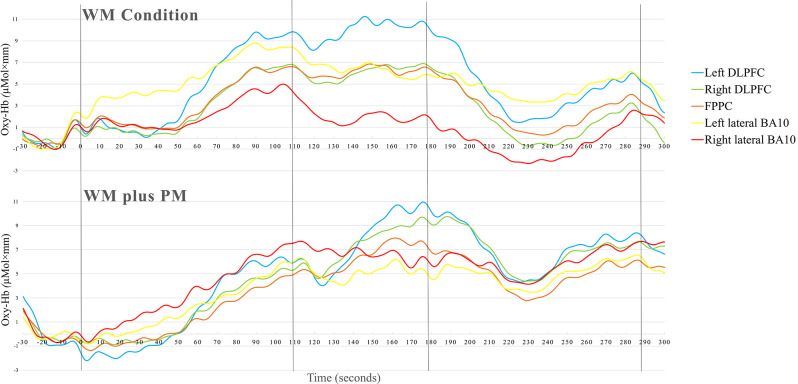
Time course of the grand averaged (oxy-Hb) changes in WM condition and WM plus PM task.

Table 3 summarizes the means and *SDs* of fNIRS data for the WM and WM Plus PM conditions in the five ROI. To compare the Δoxy-Hb in WM Condition and WM plus PM Conditions in the left DLPFC, right DLPFC, FPPC, left lateral, and right lateral BA10, a series of one-tailed paired *t*-tests were conducted. Upon visual inspection, overall activation was observed in all ROI. Except for the left lateral BA10, higher activation was observed for the WM plus PM Condition compared to the WM Condition across all ROIs. See Figure 4 for the grand mean time series of all ROIS.

**Table 3:** Descriptive statistics of oxy-Hb (μMol × mm) in ROIs during WM condition and WM plus PM task (*N* = 32).

	WM Condition		WM plus PM Condition		oxy-Hb difference				
	*M*	*SD*	*M*	*SD*	*M*	*SD*	*t*_(31)_	*p*	*d*
Left DLPFC	3.59	6.31	5.30	8.07	1.71	10.49	−0.92	0.182	0.16
Right DLPFC	1.87	4.86	4.65	4.72	2.78	6.31	−2.49**	0.009	0.44
FPPC	1.87	4.49	3.61	5.41	1.74	5.52	−1.78*	0.043	0.32
Leftlateral BA10	3.77	4.83	3.67	5.75	−0.10	8.39	0.07	0.474	0.01
Right lateral BA10	4.20	5.15	4.80	5.89	0.60	7.29	−0.47	0.322	0.08

Note: DLPFC, dorsolateral prefrontal cortex; FPPC, fronto-polar prefrontal cortex; BA10, Brodmann Area 10. * and ** denote significant difference between WM Condition and WM plus PM Condition..

**Figure 5:**
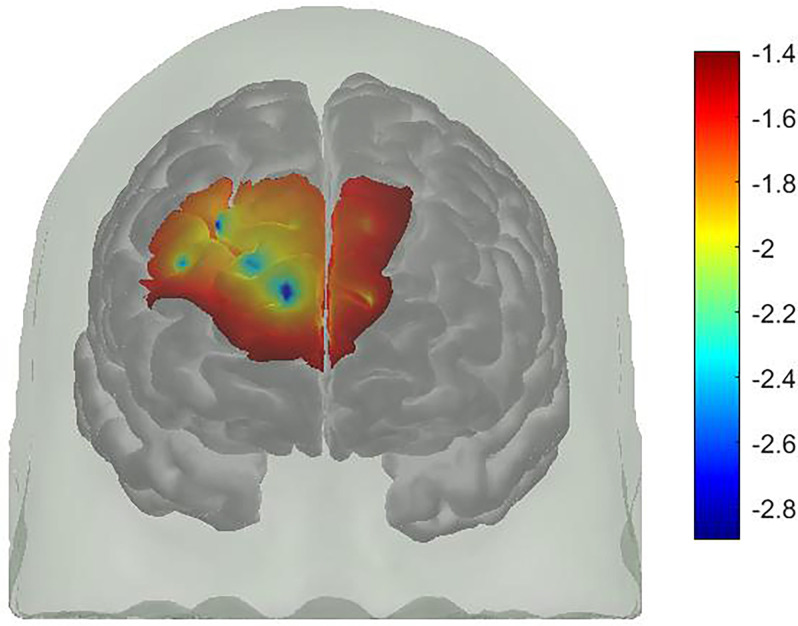
A *t*-map of oxy-Hb signal change (WM plus PM condition minus WM condition).

For FPPC, there was a significant difference between WM Condition and WM plus PM Condition, *t*_(31)_ = −1.78, *p* = 0.043, *d* = 0.32. This was a small to medium effect size, with higher oxy-Hb in WM plus PM Condition compared to WM Condition (3.61 vs. 1.87). Similarly, there was a significant difference between WM Condition and WM plus PM Condition for right DLPFC, *t*_(31)_ = −2.49, *p* = 0.009, *d* = 0.44. This was a small to medium effect size, with higher oxy-Hb in WM plus PM compared to WM Condition (4.65 vs. 1.87). For left DLPFC, the difference between WM Condition and WM plus PM Condition was not significant, *t*_(31)_ = −0.92, *p* = 0.182. *d* = 0.16. Lastly, for left lateral BA10, *t*_(31)_ = −0.10, *p* = 0.474, *d* = 0.01, and right lateral BA10, *t*_(31)_ = −0.47, *p* = 0.322, *d* = 0.08, there was no significant differences between WM Condition and WM plus PM Condition (See Figure 5).

## Discussion

The aim of this study was to develop an experimental PM task suitable to investigate neural activation during PM processes using fNIRS and to provide corroborating evidence for the neural basis of PM. Our behavioral results showed that participants were significantly slower to respond to the 2-back task in the WM plus PM Condition compared to WM Condition. However, there were no significant differences in task accuracies for the same task. fNIRS results showed that right DLPFC and FPPC showed higher activation during the WM plus PM Condition compared to the WM Condition.

Behaviorally, an interference effect for RT was observed, whereby participants were significantly slower to respond to the 2-back task in the WM plus PM Condition compared to WM Condition. In contrast, there were no significant differences in the 2-back task accuracies between the WM Condition and WM plus PM Condition. These results indicate that participants were engaged in the ongoing task and did not exclusively allocate their resources for the PM task. The increased RT may reflect the reallocation of attentional resources to the PM task. This interference effect is classic and reflects the cognitive cost of maintaining an intention while performing another task (Hicks et al., 2005; Loft et al., 2008). Thus, additional processes were required to engage in PM relative to the ongoing task alone consistent with previous studies investigating PM interference effects (Hicks et al., 2005; Cohen et al., 2011). Such interference effects are specific to the PM intention, not the overall cognitive load (Hicks et al., 2005; Cohen et al., 2011). Overall, participants’ PM performance did not show floor or ceiling effects (range: 0–10, *M* = 0.68) which supports the utility of the experimental PM task for a healthy adult sample.

The fNIRS results showed there were significant differences in neural activation of the FPPC and right DLPFC when comparing the WM Condition and WM plus PM task. That is, both regions showed higher activation in the WM plus PM condition compared to the WM condition. This finding partially aligns with a positron emission tomography study by Okuda et al. (1998), who also used a laboratory-based paradigm. They found several highly localized frontal and medial temporal activations—in the right DLPFC (BA 8 and 9), right ventrolateral PFC (BA 47), left frontal pole (BA 10), left anterior cingulate gyrus (BA 24), midline medial frontal lobe (BA 8), and left parahippocampal gyrus (BA 28) during their PM task (i.e., word repetition task) compared to the control task (i.e., word repetition task without PM targets). These findings are also congruent with previous studies finding that DLPFC activation is associated with WM processes, and thus confirms the validity of this task since it also contained a WM component (Reynolds et al., 2009). It also suggests that more cognitive effort is required for a WM plus PM task in comparison to a WM only task.

The finding that the right DLPFC was significantly more active during the WM plus PM task also partially aligns with a transcranial magnetic stimulation (TMS) study by Basso et al. (2010) investigating whether WM and PM are based on a single common system. The researchers assessed the interaction by manipulating the cognitive demands during WM and event-based PM tasks. They found that WM and PM only competed for resources at high levels of WM load since interference effects on PM were produced by TMS on the DLPFC (bilateral). Their results show that while WM and PM may share the same associative brain regions, the TMS effect produced on the task (in bilateral DLPFC) was strictly associated with PM performance. Therefore, the finding that the right DLPFC was more active during the WM plus PM task may suggest that the task load in the WM plus PM condition required participants to recruit this area to maintain accurate performance, albeit impacting RT.

In contrast, no significant differences were evident in left and right lateral BA10 activity for the PM task component compared to the ongoing WM task components. This is contrary to previous research, which has typically found the left BA10 to be associated with event-based PM (Burgess et al., 2003; Gilbert et al., 2006, 2010; McDaniel et al., 2013; Beck et al., 2014; Cona et al., 2015; Dong et al., 2019). This was not replicated in this study and thus, did not support our hypothesis. We found that left and right lateral BA10 activity was comparable between task conditions. However, this does not necessarily indicate that BA10 is not associated with PM processes. An explanation for the lack of differences in activity in BA10 may be due to the similarity between the WM Condition and WM plus PM Conditions (i.e., the PM task was embedded in the WM task). Therefore, the overall cerebral activity observed in all PFC areas, including BA10 may have been suppressed by the WM only processes. In fact, other studies typically only compared hemodynamic responses within the PM task itself (e.g., Dong et al., 2019) rather than between related tasks. In addition, more recent fNIRS studies (Pinti et al., 2015; Dong et al., 2019) have employed either immersive VR or naturalistic paradigms to study PM using fNIRS. Thus, these task differences may account for the absence of additional BA10 activity in the present study.

These findings are also in line with previous work investigating fMRI correlates of PM processes in non-focal vs. focal tasks (McDaniel et al., 2013; Cona et al., 2015). McDaniel et al. (2013) found that there are two distinct routes in PM processing. The first route is active during the retrieval phase and involves ventral parietal regions, and insular and cingulate cortices. This route was interpreted to mediate bottom-up processes. While the second route includes activity in dorsal frontoparietal regions, including DLPFC and the superior parietal lobe. This was interpreted to be involved in top-down monitoring processes, typically recruited during the maintenance phase, and only for non-focal PM cues. However, due to the limitations in the scope of fNIRS to measure subcortical activation and cortical-subcortical connectivity, it is not possible to speculate which routes were most active when participants completed the WM plus PM task.

Lastly, the fNIRS results suggest that there may be a neural adaptation to the WM task (ongoing task). This explanation can be supported by the grand mean time series, which showed that PFC activation did not return to baseline before commencing the WM plus PM task. Another possibility is that the WM plus PM condition task load was not difficult enough for the sample of healthy young adults. However, this is also unlikely as an interference effect for RT was observed, with no differences in WM ongoing task accuracies. Participants were also not scoring at a ceiling level for the PM task component. Other studies examining WM load and neural adaptation (Liu et al., 2015) have also observed reduced brain activations over time, and have concluded that they are most likely an exclusive feature of studies with short practice times (Klingberg, 2010). Since the WM plus PM condition only differs by an additional embedded task, it is possible that participants treated the WM Condition as practice, and consequently adapted to the WM plus PM task due to task order.

### Implications

These findings add to a body of literature investigating the neural underpinnings of PM. The aims of this study were addressed—an appropriate dual-task PM paradigm was developed and found to be suitable to measure PM performance. Although we could not differentiate levels of FPPC and lateral BA10 activation between tasks, these findings still corroborate the findings of previous studies indicating that the PFC (including medial BA10) was involved in PM task performance. In comparison to the findings of other studies, it is apparent that differences in PM experimental paradigms could result in associations with different regions. Currently, there is more evidence showing that studies employing VR and naturalistic tasks (Pinti et al., 2015; Dong et al., 2019) have replicated fMRI findings. Less research has been conducted on fNIRS using experimental laboratory paradigms. Lastly, these results suggest that the right DLPFC and FPPC may also be involved in PM processes. However, differences in cognitive neuroscience methods may produce different patterns of activations, for instance, a TMS study also showed that the DLPFC is associated with PM processes (Basso et al., 2010), while fMRI and PET studies have typically found BA10 along with other highly localized frontal areas to be associated (Okuda et al., 1998; Burgess et al., 2003).

### Limitations and Suggestions for Future Research

Notwithstanding the novel findings, some limitations must be acknowledged. First, the lack of differences in ROI across the two task conditions may be attributed to the lack of spatial resolution with the fNIRS methodology. For instance, as a large area of BA10 was chosen, it is possible that a more focal region would elicit more differences in cerebral activity. Moreover, the region categorized as FPPC also overlaps with some of BA10. However, this could also be a spatial limitation. Future studies should undertake a more detailed subdivision of BA10 using other methods such as fMRI or PET. Unlike fMRI studies which are able to explore deeper regions, a limitation of fNIRS is its focus only on superficial areas of the cortex (Quaresima et al., 2012). Moreover, activation was observed across the entire PFC, even in BA10. Hence, it was not possible to detect differences in activation between very similar tasks (WM Condition vs. WM plus PM) to observe additional activity associated with PM processes. It is recommended that future studies design a task that is different enough to detect PM activity while also being comparable to the non-PM task. This could be a 0-back task, or an alternative matched task. Importantly, these lack of differences may also be due to task order. Future studies should consider counterbalancing task order with longer breaks between tasks to ensure cerebral activity returns back to baseline. Due to the highly localized areas involved with PM, future fNIR studies should aim to investigate the older adult or clinical populations who typically show PFC changes to assess PM.

## Conclusion

This study designed a laboratory-based PM task to examine the neural correlates of PM using fNIRS. Results demonstrated the feasibility of using this PM task to investigate cerebral activity using fNIRS. We found higher cerebral activity within the FPPC and right DLPFC during the WM plus PM compared to the WM Condition suggesting that these areas are involved in PM processes in an experimental event-based PM task. However, these findings should be interpreted with caution due to the infancy of fNIRS research in PM. Further research using fNIRS investigating PM using different experimental paradigms is warranted.

## Data Availability Statement

The raw data supporting the conclusions of this article will be made available by the authors, without undue reservation.

## Ethics Statement

The studies involving human participants were reviewed and approved by Griffith University. The patients/participants provided their written informed consent to participate in this study.

## Author Contributions

YWK, MY, and DS designed the study and analyzed data. YWK wrote the manuscript. All authors provided critical revisions to the manuscript and contributed to the conception of the work. All authors contributed to the article and approved the submitted version.

## Conflict of Interest

The authors declare that the research was conducted in the absence of any commercial or financial relationships that could be construed as a potential conflict of interest.

## Publisher’s Note

All claims expressed in this article are solely those of the authors and do not necessarily represent those of their affiliated organizations, or those of the publisher, the editors and the reviewers. Any product that may be evaluated in this article, or claim that may be made by its manufacturer, is not guaranteed or endorsed by the publisher.
